# Arrhythmia Inducibility in the CAVB Dog Model, A Critical Analysis on Underlying Factors

**DOI:** 10.1007/s12012-025-10033-3

**Published:** 2025-06-24

**Authors:** Joanne J. A. van Bavel, Henriëtte D. M. Beekman, Marien J. C. Houtman, Marc A. Vos, Marcel A. G. van der Heyden

**Affiliations:** Department of Medical Physiology, Division of Heart and Lungs, University Medical Center Utrecht, Utrecht, the Netherlands

**Keywords:** AV block dog model, Remodeling, Torsade de pointes, Arrhythmia predisposition

## Abstract

The dog with chronic atrioventricular block (CAVB) combines a number of risk factors associated with Torsade de Pointes (TdP) arrhythmias. Nevertheless, approximately 33% of the animals are resistant to dofetilide-induced TdP arrhythmia. Of a group of 78 experimentally identical CAVB dogs, we compared TdP inducible vs. non-inducible animals for a set of basic, and cardiac electrical and mechanical parameters. Body weight, but not sex or age, is associated with TdP inducibility. Of the cardiac parameters, longer ventricular repolarization duration and increased contractility at baseline are associated with dofetilide-induced TdP arrhythmias. Differences in cardiac parameters disappeared upon dofetilide infusion. We discuss that prolonged repolarization and increased contractility may be early indications of calcium-mediated early after depolarization that may develop into TdP arrhythmias.

## Introduction

The baseline prevalence of cardiac rhythm abnormalities is > 2% [1]. In humans, several well-established factors increase the risk for QT prolongation and Torsade de Pointes (TdP) arrhythmias, of which main contributors are female sex, electrolyte imbalance (in particular hypokalemia), age, genetic predisposition, congestive heart failure, and ischemia [2]. Moreover, many widely used medications can induce or exacerbate arrhythmias and much on its underlying mechanism remains unknown [3]. Therefore, an animal model with high and reproducible inducibility rates for ventricular arrhythmias is deemed necessary to test pro- and antiarrhythmic properties of drugs but also new device strategies.

The dog with chronic complete atrioventricular block (CAVB) is already in use for decades as a successful model in cardiac ventricular arrhythmia research [4, 5]. Many pro- and antiarrhythmic drugs and new device properties were evaluated, most often demonstrating congruence with their actions on the heart in humans [6]. The AV block-induced bradycardia causes cardiac adaptation occurring in the weeks succeeding [7] in order to maintain cardiac output as physiologically demanded. Increased neurohormonal levels and calcium handling alterations [8–11] enhance the contractility, which are slowly taken over on a structural level in the form of biventricular hypertrophy [12] without increased fibrosis formation [8]. Moreover, electrical remodeling includes diminished repolarizing I_Kr_ and I_Ks_ densities [13] and is associated with QT prolongation and a reduced repolarization reserve. These outcomes are known proarrhythmic parameters [14] and predispose the animals for drug-induced arrhythmia. Hence, a combination of bradycardia, anesthesia, and infusion of the I_Kr_ blocker dofetilide, results in TdP arrhythmias in approximately 75% of the animals, which is reproducible when tested again a few weeks later [4].

Although the high incidence of drug-induced ventricular arrhythmia makes it a useful model, it is still incomprehensible which factors or combination thereof distinguishes “inducible” animals (≥ 3 TdP arrhythmias in the 10-min testing period) from “non-inducible” animals (≤ 2 TdP arrhythmias). Namely, all animals have undergone the same experimental treatments, are of mixed breeds, and even between littermates inducible and non-inducible animals are apparent. In this sense, the model mirrors several human pathological conditions in which only a proportion of the affected people encounter cardiac arrhythmias, while all have a combination of risk factors [14].

Most published studies using CAVB dogs present only small experimental groups (3–12 animals) [6], hampering matching presented risk factors to occurrence of cardiac arrhythmia. Moreover, methodology is slightly different between laboratories or change over time, adding to the difficulties in retrospective meta-analysis. Since we have performed many experiments over the last 10 years in CAVB dogs with identical methodology, we decided to explore our database for currently unpublished general characteristics and cardiac responses of the CAVB dog model in an attempt to answer the question “what makes a CAVB dog inducible for TdP arrhythmias?”.

## Methods

### Animals

In this analysis, a total of 78 Marshall Mongrel™ dogs (Marshall, NY, USA) were included that had undergone TdP inducibility testing between the years 2010 and 2020. Animal care and experimentation were approved by the Committee for Experiments on Animals of Utrecht University and were in accordance with the Directive 2010/63/EU of the European Parliament and the Dutch law on animal experimentation. Full experimental details including notifications on ARRIVE guidelines can be found in the associated publications [7, 15–21].

### General Protocol for TdP Inducibility Testing

AV block was induced by radiofrequency ablation of the His-bundle. Dogs then underwent remodeling at IVR for > 2 weeks (on average CAVB4 ± 2 weeks for both inducible and non-inducible animals), and animals were tested for TdP inducibility with dofetilide (0.025 mg/kg, i.v.) after anesthesia induction with sodium pentobarbital (25 mg/kg i.v.) and maintenance with isoflurane (1.5%). The predefined proarrhythmic period in testing CAVB dogs is within 10 min after the start of dofetilide or until the first TdP event occurs. CAVB dogs that show ≥ 3 episodes of TdP in the 10 min after starting dofetilide are considered inducible.

### Data Analysis

Analyses solely included results from experiments with an identical methodology (dog breed and age (1–2 years), generation of AV block, remodeling at idioventricular rhythm (IVR), anesthesia, dofetilide infusion regimen, housing, animal handling, researcher). Arrhythmic events during the predefined arrhythmic period were scored as follows: single ectopic beats with 2 points, multiple ectopic beats with 3–5 points and self-terminating TdP arrhythmias with 6–49 points. When defibrillations were necessary to end TdP arrhythmias, these events were scored with 50, 75, 100 points for one, two or three defibrillations, respectively. The arrhythmia score was based on the average of the three highest scored arrhythmic events. A detailed overview of the data analyses is described by Van Bavel et al., [20].

### Statistics

Data are presented as mean ± SD. Data were analyzed using an unpaired Students *t*-test or a one-way or two-way analysis of variance (ANOVA). Statistical analyses were performed using Graphpad Prism (version 9.3.1, Graphpad Software, San Diego, USA). A value of *p* < 0.05 was considered statistically significant.

## Results

### General Characteristics vs. Inducibility

**Fig. 1:**
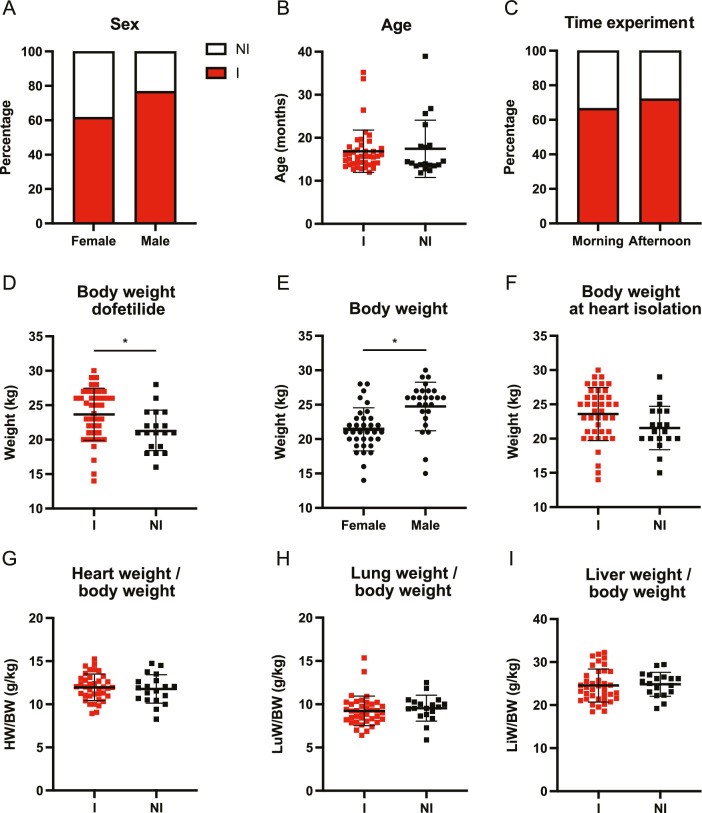
Arrhythmia inducibility per general characteristic of chronic AV block dogs. **A** Sex (female *n* = 34, male *n* = 27), **B** age, **C** time of dofetilide experiment (morning *n* = 43 and afternoon *n* = 18), **D** body weight during dofetilide experiment, **E** body weight of female and males, **F** body weight at heart isolation timepoint, **G** heart weight/body weight (HW/BW), **H** lung weight/body weight, and **I** liver weight/body weight at heart isolation timepoint. I = inducible, and NI = non-inducible. Mean ± SD, two-way ANOVA and unpaired students t-test, * refers to *p* < 0.05

With regard to sex, inducibility was slightly higher in males but no significant differences were observed. 62% of the females and 77% of the males were inducible (Fig. 1A) and thus the selected group represented the known fraction of inducible animals of approximately 75%. Moreover, no age difference was observed between the two groups (16.9 ± 4.9 vs. 17.4 ± 6.8 months for inducible and non-inducible animals, respectively) (Fig. 1B). The time of the experiment—in the morning or in the afternoon—had no effect on the inducibility outcome (Fig. 1C). On a group level, body weight was significantly different between inducible and non-inducible dogs (Fig. 1D), but in general male dogs were heavier than females (Fig. 1E). The body weight at heart isolation was lower for non-inducible dogs, though not significant (Fig. 1F) and the relative heart weight (heart weight *vs*. body weight, *i.e.* an indication of cardiac hypertrophy) was similar between groups (Fig. 1G). The same holds true for relative lung and liver weight (Fig. 1H, I) indicating no explicit implications for heart failure. Furthermore, body weight difference between the inducible and non-inducible dogs is rather small, and a substantial overlap between the two groups is apparent. Since all animals were of mixed breed and obtained from the same source (Marshall, NY, USA), no analysis with respect to these factors could be performed. We also do not have complete knowledge on littermates in each group of animals though inclusion of littermates within experimental setups was certainly limited.

### Electrophysiological Parameters vs. Inducibility

Of all ECG parameters analyzed from remodeled CAVB dogs before dofetilide was added (baseline), only those representing ventricular repolarization intervals (QT, QTc, JT, and JTc) are different between inducible and non-inducible animals (Table 1). This is, QTc and JTc are approximately 70 ms longer in inducible dogs. Monophasic action potentials (MAPs) measured locally in the left and right ventricle (LV and RV, respectively), show no such difference. Beat-to-beat variation of repolarization duration quantified by short term variability (STV) in baseline conditions, either from the LV or RV, shows an increased trend in the inducible animals.

**Table 1:** Electrophysiological and contractile parameters in inducible and non-inducible chronic AV block dogs at baseline and during dofetilide

Parameter	Baseline		Dofetilide		n (I, NI)
	Inducible	Non-inducible	Inducible	Non-inducible	
RR	1405 ± 280	1295 ± 157	1550 ± 294*	1510 ± 191*	40, 18
PP	543 ± 74	574 ± 68	635 ± 98*	704 ± 127*^	28, 15
QRS	95 ± 19	91 ± 17	96 ± 21	96 ± 17	39, 18
QT	443 ± 75	364 ± 40^	613 ± 102*	607 ± 69*	40, 18
QTc	407 ± 68	338 ± 34^	565 ± 87*	562 ± 67*	40, 18
JT	347 ± 75	272 ± 36^	520 ± 98*	511 ± 61*	39, 18
JTc	312 ± 68	246 ± 31^	473 ± 83*	467 ± 59*	39, 18
LV MAP	306 ± 50	288 ± 20	479 ± 118*	485 ± 98*	27, 9
RV MAP	260 ± 37	250 ± 29	361 ± 99*	400 ± 78*	23, 10
Δ MAP	41 ± 28	35 ± 28	123 ± 86*	80 ± 72	20, 9
LV STV	1.70 ± 1.24	0.80 ± 0.42	3.57 ± 1.92*	2.54 ± 1.04*	26, 9
RV STV	1.25 ± 0.99	0.76 ± 0.18	2.99 ± 1.70*	3.79 ± 2.41*	15, 7
LVdP/dt_max_	2047 ± 416	1368 ± 205^	2724 ± 520*	2474 ± 554*	10, 5
LV EDP	11 ± 6	8 ± 5	12 ± 5	9 ± 3	13, 4
LV ESP	91 ± 8	85 ± 11	100 ± 7*	102 ± 9*	13, 4

Data presented as mean ± SD. The sample size for inducible (I) and non-inducible (NI) dogs is presented in the last column. Electrophysiological data in ms, LVdP/dt_max_ in mmHg/s, and LV EDP and LV ESP in mmHg. Two-way ANOVA, with Bonferroni’s multiple comparisons test. *p < 0.05 compared to baseline, and ^*p* < 0.05 compared to inducible dogs

Dofetilide infusion lengthens repolarization parameters, but more importantly in this respect, eliminates the repolarization differences between inducible and non-inducible CAVB dogs. Similar effects are seen with respect to STV. Only the PP-interval is significantly different between inducible and non-inducible animals, *i.e.* atrial rate is lower in non-inducible animals. In conclusion, a longer repolarization duration at baseline correlates with TdP inducibility, whereas the arrhythmia inducing trigger, dofetilide, eliminates these differences.

### Contraction Parameters vs. Inducibility

In a subset of the dogs, contraction parameters from the LV have been recorded. LV end systolic pressure (ESP) is similar between inducible and non-inducible animals in baseline and dofetilide increases LV ESP in both groups (Table 1). No differences can be observed in LV end diastolic pressure (LV EDP), either between inducible *vs*. non-inducible, neither for baseline *vs.* dofetilide. However, the maximal rise in ventricular pressure (LVdP/dt_max_) is higher in inducible animals at baseline, and whereas dofetilide increases maximal pressure rise on the one hand, it eliminates differences between inducible *vs.* non-inducible dogs.

### Arrhythmia Indication vs. Inducibility

**Fig. 2:**
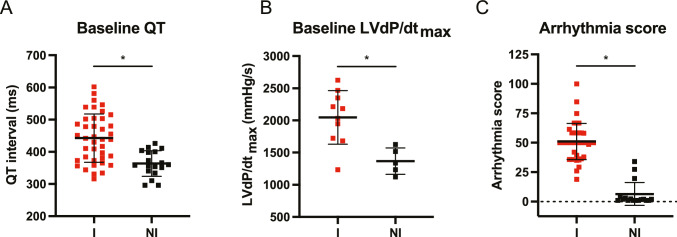
Parameters at baseline indicating arrhythmia outcome.** A** QT interval and **B** LVdP/dt_max_ at baseline for inducible (I) and non-inducible (NI) dogs. **C** Arrhythmia score reflecting the average of the three highest scored arrhythmic events after dofetilide. Mean ± SD, unpaired students t-test, * refers to *p* < 0.05

The dominant repolarization prolongation (Fig. 2A) and the enhanced contractility (Fig. 2B) of inducible dogs compared to non-inducible dogs at baseline display the indication of the arrhythmic outcome after dofetilide infusion in inducible animals. The clear difference in arrhythmia score reflects the severity of the arrhythmic events in the ten-minute time period after the onset of dofetilide infusion (Fig. 2C).

### Cellular Data

**Fig. 3:**
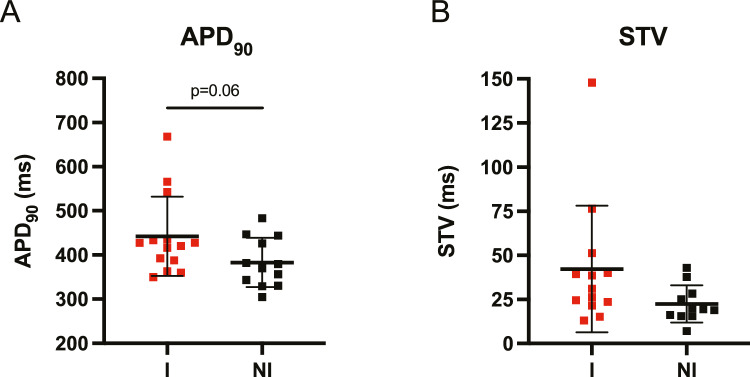
Patch clamp measurements during baseline including **A** action potential duration at 90% repolarization (APD_90_) and **B** short term variability (STV) of the APD_90_ of isolated left and right ventricular cardiomyocytes from inducible (I, *n* = 14 for APD_90_ and *n* = 13 for STV) and non-inducible (NI, *n* = 12 for APD_90_ and *n* = 11 for STV) chronic atrioventricular block dogs. Mean ± SD, unpaired Student’s t-test

Ventricular cardiomyocytes from the LV and RV of the CAVB dog have been isolated over the years. Following the final experiment in the experimental protocol, which may differ between the animals (testing of pro- and antiarrhythmic drugs or device interventions), the hearts were excised. Cell isolation was performed as described previously [22]. Under baseline patch clamp measuring conditions, there is a strong trend toward a longer action potential duration (APD) in ventricular myocytes isolated from inducible animals than from non-inducible animals (Fig. 3A). Though, the expected increase in STV of cells from inducible dogs is insignificant (Fig. 3B). No differences were found when distinguishing cardiomyocytes from the RV and LV (APD_90_: 439 ± 72 ms (LV) vs. 434 ± 128 ms (RV) for inducible dogs, and 390 ± 63 ms (LV) vs. 381 ± 64 (RV) for non-inducible dogs, STV: 37 ± 20 ms (LV) vs. 49 ± 61 ms (RV) for inducible dogs, and 25 ± 11 ms (LV) vs. 15 ± 6 ms (RV) for non-inducible dogs).

## Discussion

### Sex is Not Related to TdP Inducibility in Dogs

A number of studies indicated that in canines QTc duration and other ECG parameters do not differ between males and females [23]. This is in contrast to humans in which QTc duration is longer in females and sex is considered as a risk factor of TdP (e.g., Drew et al. 2010 and references therein) [14]. In clinical practice, dofetilide dose reductions and discontinuations occur more often in women than in men, mainly for reasons of significant QTc prolongation [24]. Importantly, our current analysis indicates that sex is not a risk factor for dofetilide-induced TdP arrhythmias in dogs.

We observed an association between body weight and TdP inducibility in dogs. In our cohort, male dogs were heavier, which may explain the apparent trend of male sex associated increase in higher inducibility. In contrast, we did not observe an association between HW/BW and inducibility. We may consider this as an absence of overt hypertrophy. We have no indications that body fat/muscle distribution changes when animals are heavier, and therefore pharmacokinetics are expected not to be affected by absolute body weight.

### Baseline Contractility in Inducible Dogs, a Reflection of Calcium Mediated Arrhythmogenesis?

In response to the permanent AV block, cardiac ventricular remodeling occurs in different phases [4, 6]. The main mechanism of TdP initiation is through the formation of early after depolarizations [25]. These result from incorrectly timed cellular calcium increase during the later phases of cardiac repolarization, although the exact mechanisms and players involved have not been resolved completely [26]. Indeed, self-terminating TdPs in the CAVB dog are initiated and maintained by a focal mechanism [27, 28]. In strong support of the role of calcium are the findings that calcium antagonists addressing intracellular calcium load (e.g. flunarizine, verapamil) are among the most effective antiarrhythmics in the CAVB dog model [7, 29–31]. As calcium stands at the origin of cardiac contraction, it may indicate that the enhanced contraction parameter in inducible animals at baseline results from excessive remodeling and resulting calcium handling, which may directly link to the underlying mechanism of TdP initiation, and thus arrhythmia risk.

### Dofetilide Alleviates Differences in Electrical and Contraction Parameters Between Inducible and Non-Inducible Dogs

The cardiac parameters that distinguish inducible from non-inducible dogs at baseline, *i.e.* ECG quantifications of repolarization and LVdP/dt_max_, increase in both groups of animals in response to dofetilide. However, as the extent of increase is higher in non-inducible animals, these parameters lose their ability to distinguish between both animal groups upon dofetilide infusion. This can be interpreted as a confirmation that prolonged repolarization and increased LVdP/dt_max_ are indicative for proarrhythmic remodeling, but that these are not directly related to triggering TdPs and their maintenance in response to dofetilide.

Over the years, we investigated two different repolarization-related parameters and their association with TdP arrhythmias. Firstly, beat-to-beat variability of repolarization, quantified as STV, is a specific type of temporal dispersion of repolarization. It increases upon dofetilide-induced lengthening of repolarization where it can distinguish between inducible and non-inducible animals [32]. Here, Table 1 indicates that baseline STV values appear to be larger in inducible animals, but did not reach significance. Furthermore, a sudden increase in STV predicts, to a certain extent, a drug (dofetilide, d-sotalol, sertindole) elicited upcoming TdP [19, 32, 33]. Increased beat-to-beat variability of repolarization likely associates with EAD formation and thus TdP initiation. Though, it should be mentioned that this parameter is sensitive to accurate determination of the end of the MAP signal and therefore demands a proper acquisition and use of cardiac signals. Secondly, spatial dispersion of repolarization (SDR), that can be measured in diverse orientations in the ventricular free walls, e.g. cubic dispersion [34], quantifies local repolarization heterogeneity. SDR has no predictive value in baseline but can distinguish between inducible and non-inducible animals upon dofetilide infusion [34]. Increased SDR was found to be associated with perpetuation of non-self-terminating TdPs [35]. In a previous study using a smaller group of animals and different anesthesia (halothane *vs.* isoflurane), QTc at baseline was also larger in inducible dogs, and upon dofetilide infusion QTc prolonged in both groups as expected, but QTc remained longer in inducible animals *vs.* non-inducible animals [32].

### Study Limitations

Inclusion of parameters Tpeak-Tend and LVdP/dt_min_ would have given additional and valuable insight into transmural dispersion of repolarization and calcium handling during the diastolic phase, respectively. However, this study was dependent on results from previous measurements (which did not include Tpeak-Tend and LVdP/dt_min_) originating from a database between the years 2010 and 2020 and therefore these parameters could not included. This also accounts for the measurement of plasma electrolyte levels.

### Future Perspectives

The dog is not useful for investigating sex differences in arrhythmia inducibility. On the other hand, it can be advantageous as sex is not a confounding factor in arrhythmia research using this species. The number of data has to be increased and we encourage other research teams using the CAVB dogs, albeit with somewhat different methodology, to use their database in similar analyses. This also holds for analyses among different TdP models to optimize risk assessment for new drug compounds [36]. More intelligent methods, e.g. machine learning-based analytics of multiple parameters may be useful to enable prediction of animals at risk for dofetilide-induced TdP.

## Data Availability

Data will be made available on request.
